# Obstructive sleep apnea (OSA) is associated with the impairment of beta-cell response to glucose in children and adolescents with obesity

**DOI:** 10.1038/s41366-023-01257-w

**Published:** 2023-01-20

**Authors:** Giuseppina Rosaria Umano, Alfonso Galderisi, Francesca Aiello, Mariangela Martino, Ornella Camponesco, Anna Di Sessa, Pierluigi Marzuillo, Papparella Alfonso, Emanuele Miraglia del Giudice

**Affiliations:** 1 Department of the Woman, the Child, and General and Specialized Surgery, University of Campania Luigi Vanvitelli, Naples, Italy; 2 Department of Woman and Child’s Health, University of Padova, Padova, Italy

**Keywords:** Obesity, Diabetes

## Abstract

**Background:**

The main purpose of the study is to assess the association between obstructive sleep apnea (OSA) and insulin secretion in children with obesity.

**Methods:**

We enrolled children and adolescents who attended our pediatric clinic because of obesity and OSA. Glucose homeostasis was assessed through standard 2-h oral glucose tolerance test (OGTT). Nocturnal cardio-respiratory polygraphy was performed for OSA diagnosis. Twenty-two patients underwent a 3-h OGTT to investigate insulin secretion and sensitivity through the oral-minimal model.

**Results:**

seventy-seven children and adolescents were included in the study. Based on OSA severity, the cohort was divided into three groups (29 mild, 29 moderate, and 19 severe OSA). The group with mild OSA showed lower levels of 30-min glucose (*p* = 0.01) and 60-min glucose (*p* = 0.03), and lower prevalence of elevated 1-h glucose (10.4% versus 44.8% in moderate and 31.6% in severe OSA, *p* = 0.01). The odds for elevated 1-h plasma glucose was 6.2-fold (95%CI 1.6–23.4) higher in subjects with moderate and severe OSA compared to mild OSA (*p* = 0.007) independent of confounders. Spearman correlation test revealed a positive correlation between 30-min plasma glucose and apnea-hypopnea index (AHI, *r* = 0.31, *p* = 0.01), oxygen desaturation index (ODI, *r* = 0.31, *p* = 0.009), and mean desaturation (*r* = 0.25, *p* = 0.04). The 3-h OGTT study included 22 participants (7 mild, 9 moderate, and 6 severe OSA). The group with mild OSA showed a higher dynamic, static, and total insulin secretion compared to those with moderate and severe OSA (*p* < 0.0001, *p* = 0.007, *p* = 0.007, respectively). AHI was significantly correlated to dynamic insulin secretion (*r* = −0.48, *p* = 0.02).

**Conclusions:**

OSA might impair beta-cell function reducing the pool of promptly releasable insulin in children and adolescents with obesity, in the absence of an effect on insulin sensitivity.

## Introduction

Obstructive sleep apnea (OSA) affects up to 40% of children and adolescent with obesity and the risk for OSA is directly correlated with obesity severity [1–3]. Several studies have pointed out that OSA per se might impair glucose homeostasis and lead to prediabetes and type 2 diabetes [4–6]. In fact, oxygen desaturation has been suggested to be a leading cause of glucose metabolism derangement and insulin-resistance independent of confounding factors [4, 5].

Despite this evidence, the pathophysiologic underpinnings of the association between OSA and glucose metabolism remain unclear, especially in pediatric groups. Mechanistic animal studies suggest that chronic intermittent hypoxia, which is the clinical consequence of OSA, may induce pancreatic beta-cell damage and lowers glucose-induced insulin secretion [7–10]. It remains unclear if this mechanism is also in place in humans and in particular in children with obesity. To fill this gap of knowledge, we enrolled 77 children and adolescents with obesity and performed a 2-h oral glucose tolerance test (OGTT) and a cardio-respiratory polygraphy. To derive more granular information on insulin secretion, in a subgroup of 22 children and adolescents we performed a prolonged 3- h OGTT and calculated the beta cell response to circulating glucose through an oral minimal model. To the best of our knowledge, this is the first study to evaluate the association between insulin secretion in children and adolescents with indexes of OSA.

## Materials and methods

We enrolled children and adolescents with obesity from the outpatient obesity clinic of the University of Campania “Luigi Vanvitelli”. Children were eligible if they had a body mass index (BMI) ≥ 95th for age and sex according to reference charts [11] and presented suspected OSA based on sleep questionnaire screening [12]. Patients using medications affecting glucose or lipid metabolism, diagnosed with syndromes, endocrinopathies, and genetic forms of obesity were excluded. Written informed consent from parents and children assent was obtained before any procedure. The study was conducted according to the criteria set by the Declaration of Helsinki. The Institutional Review Board of the University of Campania Luigi Vanvitelli approved the study (protocol n. 834/2016). Written informed consent was obtained before any procedure. All participants underwent an anthropometric evaluation, overnight cardiorespiratory polygraphy, and a standard 2-h OGTT. In addition, a subgroup of patients underwent a 3-h OGTT to better evaluate insulin secretion by using the oral minimal model (OMM).

### Clinical examination

Weight was measured by a balance beam scale; the child wearing undergarments. Height was measured by a Harpenden stadiometer. BMI was calculated as weight (kg)/height^2^ (*m*^2^). Z-score BMI was calculated with the lamba-mu-sigma method [13] according to reference charts to standardize BMI value according to age and gender [11]. Pubertal status was defined according to Tanner stage evaluating breast development in girls and testicular volume and genitalia development in boys [14]. Prepubertal boys and girls were defined as Tanner I, post-pubertal boys and girls were defined as Tanner III.

### Biochemical assessment

After informed consent, a blood sample was drawn after an overnight fast. The serum was frozen at −20 °C until analyzed. Triglycerides, total cholesterol, LDL-cholesterol, and HDL-cholesterol levels were determined by an enzymatic colorimetric test with lipid clearing factor (ATLAS MEDICAL, Blankenfelde-Mahlow, Germany). Serum ALT and AST were assayed using a Hitachi Analyzer (Boerhinger-Mannheim Diagnostics, Indianapolis, IN).

### Oral glucose tolerance test

After a 12-h overnight fast, children and adolescents were admitted to the Pediatric clinic of University of Campania Luigi Vanvitelli. One antecubital intravenous catheter was inserted after the local appliance of anesthetic cream for blood sampling. Flavored glucose at a dose of 1.75 g/kg weight up to a maximum of 75 g was given orally. Blood samples were obtained every 30 min for 120 min for glucose and insulin serum levels measurements. Elevated 1-h glucose was defined as 60-min glucose ≥133 mg/dL [15]. Homeostasis model assessment for insulin resistance and whole-body insulin sensitivity index (WBISI) [16] were used as measures of insulin resistance. The composite WBISI is based on values of insulin and glucose obtained from the 5-points OGTT and represents a good estimate for clamp-derived insulin sensitivity [16]. Immunoreactive insulin was assayed by IMX (Abbott Diagnostics, Santa Clara, CA). The mean intra- and inter-assay coefficients of variations were 4.7% and 7.2%, respectively.

Insulin secretion was estimated by insulinogenic index (IGI), that is a valid surrogate of beta cell function during OGTT. It is calculated as the ratio of insulin concentration at 30 min minus fasting insulin to the difference of glucose at same time [17]. Oral disposition index (ODI) was obtained by the product of WBISI and IGI [17].

### Oral minimal model

Twenty-two children and adolescents underwent a 3-h OGTT to assess the OMM. Beta-Cell responsiveness (*φ*_total_*)* was computed from glucose and c-peptide using the oral c-peptide minimal model [18]. Briefly, the model describes glucose-stimulated insulin secretion as the result of two components: a dynamic component, representing the secretion of promptly releasable insulin vesicular pool, that is proportional to the rate of glucose increase (*φ*_dynamic_) and a static component, deriving from new insulin synthesis, this latter described by a static responsivity index (*φ*_static)_. The dynamic component describes the early insulin release due to the resident insulin pool of beta-cells and it is proportional to the rate of glucose change, therefore influencing the early glucose raise during the OGTT. Insulin sensitivity (SI) was estimated from glucose and insulin measurements during the 3-h OGTT using the oral glucose minimal model. The *β*-Cell responsiveness in the contest of insulin sensitivity was described by the product of *φ*_total_ × SI and defined through the disposition index (DI) [18,19].

### Pediatric Sleep Questionnaire

The Pediatric Sleep Questionnaire (PSQ) [12] scores 22 items that investigate the snoring frequency, loud snoring, observed apneas, difficulty breathing during sleep, daytime sleepiness, inattentive or hyperactive behavior, and other pediatric OSA features, each previously shown to correlate with OSA confirmed by cardiorespiratory polygraphy in referred children [12]. The items are divided into nocturnal, daytime, and cognitive symptoms. Each item is scored as present, absent, or unknown. The score is calculated by dividing the number of symptoms that are present by the total number of symptoms that are present or absent (present/present + absent); any questions that are not answered (unknown) are not included in the calculation. The score can vary from 0 to 1. Previous data suggest that a cutoff value of 0.33 is most effective in identifying pediatric OSA [12,20]. All children and adolescents presenting a PSQ score ≥0.33 underwent a cardiorespiratory polygraphy.

### Cardiorespiratory polygraphy

Sleep recording started at child’s usual bedtime and continued until the spontaneous awakening in the morning. Recordings were performed with Embletta® Gold device (Embla Systems Inc, Ontario, Canada) including the following sensors: nasal cannula pressure, thermistor, pulse oximeter, thoracic and abdominal respiratory inductance plethysmography, and electrocardiogram. Visual scoring of respiratory events was performed according to American Academy of Sleep Medicine (AASM) scoring criteria 2012 for children [21]. This abbreviated polygraphy without electroencephalographic, electro-oculographic or electromyographic leads has previously been demonstrated to be an accurate tool for the detection of OSA [22]. An obstructive apnea was scored if there was >90% fall in nasal pressure transducer for >90% of the entire event, the event lasted ≥2 breaths, and there was continued or increased respiratory effort. A mixed apnea was scored when there was absent respiratory effort during one portion of the event and the presence of inspiratory effort in another portion, regardless of which portion comes first. A central apnea was scored if there was absent respiratory effort for the entire event, and either the event lasted for >20 s or lasted ≥2 breaths and was associated with a ≥ 3% oxygen desaturation. A hypopnea was scored if there was a ≥ 30% reduction in amplitude of the nasal pressure transducer, the event lasted for ≥2 breaths and was associated with a ≥3% oxygen desaturation. The final apnea-hypopnea index (AHI) was determined by dividing the total number of apneas and hypopneas by the number of hours of total sleep time. A value of AHI of >1 was indicative of OSA. The ODI (defined as the number of drops in oxygen saturation ≥3% per hour of sleep time) was also calculated during the study analysis. Mean oxygen saturation (SpO2), mean oxygen desaturation (mean oxygen drop of desaturation events), and lowest oxygen saturation (Nadir) were recorded. OSA severity was scored according to pediatric criteria: mild OSA for 1 < AHI ≤ 5, moderate OSA 5>AHI < 10, severe OSA AHI ≥ 10 [21].

### Statistical analysis

Continuous variables were checked for normality with Kolmogorov-Smirnov test. Difference for continuous variables were assessed with ANOVA test or Kruskall-Wallis test as appropriate. Post-hoc analysis for multiple comparisons has been performed with Dunn test. Fisher exact test or Chi-square test were performed for difference in categorical variables. Univariate logistic regression analysis was performed to assess the risk of presenting elevated 1-h plasma glucose according to OSA severity. Multiple logistic regression analysis was performed with age, z-score BMI, and pubertal stage as covariates. Spearman correlation test was performed to investigate the correlation between respiratory sleep parameters and OGTT-derived parameters. Generalized linear models (GLM) were performed to investigate the association between each respiratory parameter and each OGTT parameter adjusting for age, sex, Tanner stage, and z-score BMI. Data are expressed as median (interquartile range, IQR) or frequencies. All the analyses have been performed using SAS® on Demand for Academics (SAS Institute Inc., Cary, NC).

## Results

### OSA severity and glucose homeostasis during 2-h OGTT

A total of 77 children and adolescents (41 males) were included, with a median age of 11.3 ± 2.7 years, mean z-score BMI of 3.53 ± 0.69. Among them, 29 subjects (37.7%) presented with mild OSA, 29 (37.7%) showed moderate OSA, and 19 (24.6%) with severe OSA. Differences between these three groups are shown in Table 1.

**Table 1:** Anthropometric, biochemical, and polysomnographic characteristics of children with mild, moderate, and severe OSA.

	Mild OSA (*N* = 29) (%, or median and IQR)	Moderate OSA (*N* = 29) (%, or median and IQR)	Severe OSA (*N* = 19) (%, or median and IQR)	*p* for difference
Gender (M)	47.7	55.2	61.1	0.60
Age (years)	12.8 (10.1–14.3)	11.2 (9.6–13.7)	9.9 (8.4–12.3)	0.08
Tanner Stage (I/II/III, %)	31/27.6/41.4	47.8/34.8/17.4	33.3/15.8/11.1	0.11
z-score BMI (years)	3.4 (2.9–3.7)	3.6 (3.0–3.8)	3.6 (3.3–4.3)	0.17
Fasting Glucose (mg/Dl)	69 (75–80)	73 (68–81)	71 (66–76)	0.74
Fasting Insulin (mcU/mL)	14.1 (11.3–27.0)	18.5 (11.0–30.7)	18.5 (11.1–32.9)	0.68
30-minutes glucose (mg/dL)	118 (113–125)	131 (117–137)	130 (111–137)	**0.01**
60-minutes glucose (mg/dL)	116 (99–125)	135 (112–152)	123 (96–148)	**0.04**
90-minutes glucose (mg/dL)	112 (96–132)	111 (101–125)	106 (96–129)	0.83
120-minutes Glucose (mg/dL)	107 (96–126)	118 (103–124)	101 (97–115)	0.32
120-minutes Insulin (µU/mL)	62.9 (35.0–135.0)	106.5 (58.0–171.8)	74.3 (22.7–106.8)	0.23
HOMA-IR	2.9 (2.0–5.2)	3.6 (1.7–5.7)	3.4 (1.9–5.6)	0.81
WBISI	3.6 (2.2–5.8)	2.9 (1.7–4.0)	2.2 (1.9–5.8)	0.15
IGI	1.6 (1.2–2.7)	2.0 (1.0–2.9)	1.6 (0.9–4.0)	0.95
DI	6.44 (3.7–9.4)	4.9 (3.0–7.8)	5.6 (3.3–8.1)	0.53
HDL-cholesterol (mg/dL)	40 (33–46)	39 (33–48)	39 (36–46)	0.89
Triglycerides (mg/dL)	108 (91–130)	122 (97–159)	92 (83–132)	0.36
LDL-cholesterol (mg/dL)	90 (71–114)	92 (67–120)	105 (85–121)	0.53
ALT (U/L)	23 (19–35)	22 (20–31)	32 (23–48)	0.09
AST (U/L)	21 (18–27)	22 (19–28)	26 (20–33)	0.31
AHI (events/hour)	3.3 (2.6–4.0)	6.4 (5.3–7.3)	21.5 (15.7–35.4)	**<0.0001**
ODI (events/hour)	2.2 (1.5–3.8)	5.3 (3.3–6.8)	18.8 (15.8–47.8)	**<0.0001**
Mean SpO2 (%)	96.5 (95.8–97.5)	96.9 (96.1–97.4)	95.0 (91.6–96.3)	**<0.0001**
Mean Desaturation (mean oxygen drop during events)	3.6 (3.4–4.1)	3.9 (3.6–4.3)	4.6 (4.0–7.3)	**0.0002**
Saturation nadir (%)	90 (89–93)	90 (88–92)	80 (67–92)	**<0.0001**

AHI apnea/hypopnea index, *ALT* alanine aminotransferase, *AST* aspartate aminotransferase, *HOMA-IR* homeostasis model assessment for insulin resistance, *IGI* insulinogenic index, *DI* disposition index, *ODI* oxygen desaturation index, *WBISI* whole body insulin sensitivity index.

Data are expressed as median (interquartile range). Significant *p* values are reported in bold.

The three groups did not differ in age, z-score BMI, gender, and pubertal stages. Moderate and severe OSA group had significantly higher AHI and ODI levels (*p* < 0.0001). Moreover, they presented higher mean desaturation levels (*p* = 0.0002), lower mean SpO2 (*p* < 0.0001), and nadir (lowest overnight saturation) values (*p* < 0.0001). Post-hoc analyses confirmed the significance of these differences among the three groups (Supplementary Table 1). Severe OSA group showed a trend toward higher ALT levels compared to the other two groups (*p* = 0.09). With regards to glucose homeostasis, we observed a significant difference for 30-min glucose and 60-min glucose among the three groups (*p* = 0.01 and *p* = 0.04, respectively). Post-hoc analyses showed that mild OSA group had lower 30-min and 60-min glucose levels compared to moderate OSA (*p* = 0.01 and *p* = 0.04), whereas no difference was observed in the other comparisons (Supplementary Table 1).

Prevalence of elevated 1-hour glucose was significantly lower in children and adolescents with mild OSA (10.4%) compared to the other groups (44.8% and 31.6%, *p* = 0.01). No difference was found in impaired fasting glucose (IFG), impaired glucose tolerance (IGT), and type-2 diabetes (T2D) prevalence (cumulative prevalence 3.4%, 3.4%, and 10.4% in mild, moderate, and severe OSA, *p* = 0.48). The odd of showing elevated 1-h plasma glucose was 6.2-fold (95% CI 1.6–23.4) higher in children and adolescents with moderate and severe OSA compared to mild OSA (*p* = 0.007). Multivariate analysis confirmed this association even adjusting for confounding factors (*p* = 0.002, Supplementary Table 2). No differences were found for insulin resistance, lipid profile, and liver enzymes between the groups.

Spearman correlation showed a positive correlation between 30-min plasma glucose and AHI (*r* = 0.31, *p* = 0.01), ODI (*r* = 0.31, *p* = 0.009), and mean desaturation (*r* = 0.25, *p* = 0.04). GLM showed a significant association between AHI (*p* = 0.003) and ODI (*p* = 0.003) with 30-min plasma glucose. Conversely, the association of mean desaturation with 30-min plasma glucose was no longer significant after adjusting for confounders.

### OSA severity and insulin secretion

The subgroup of 22 children and adolescents underwent a 3-h OGTT did not differ from the 2-h OGTT cohort for age, z-score BMI, gender, pubertal stage, and OSA severity (Supplementary Table 3).

The group included children and adolescents, of whom seven had mild OSA, nine moderate OSA, and six had severe OSA. No differences were found for anthropometric characteristics, glucose levels, insulin sensitivity, DI, and IGI (Supplementary Table 4).

**Fig. 1:**
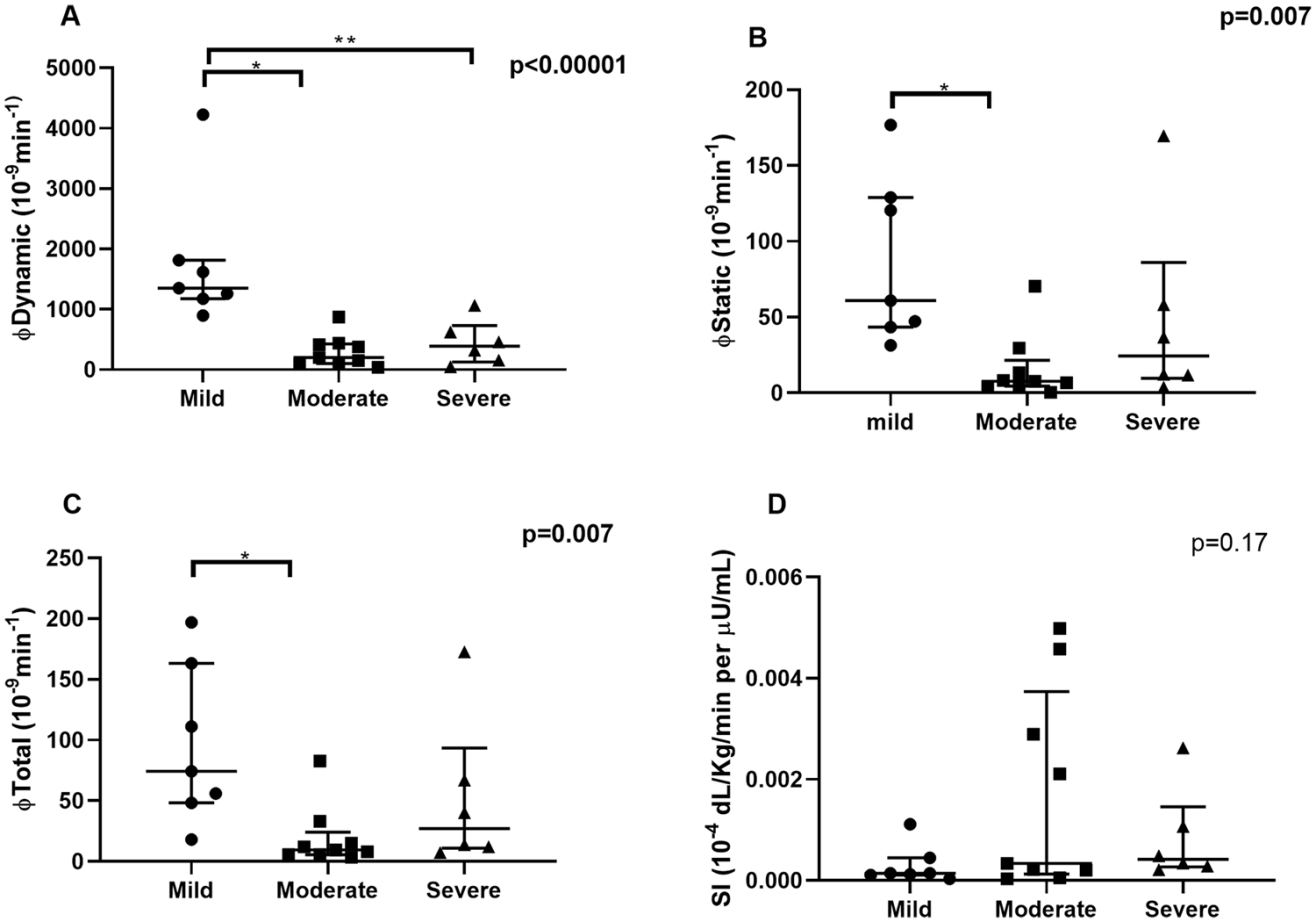
Kruskal-Wallis test and post-hoc analyses for dynamic, static, total insulin secretion, and insulin sensitivity according to obstructive sleep apnea (OSA) severity group. Mild OSA group showed higher dynamic (**A**), static (**B**), and total (**C**) insulin secretion. No differences were observed in insulin sensitivity (SI, **D**). Individual values with median and interquartile range are shown. ** indicates *p* < 0.05; * indicates *p* < 0.01.

No differences were found for insulin sensitivity (SI, *p* = 0.17, Fig. 1, panel D) and disposition index according to OMM (beta-cell function adjusted for insulin sensitivity, *p* = 0.87). Nevertheless, when beta cell responsiveness was dissected into its dynamic (*φ*_dynamic_) and static (*φ*_static_) components, as described by the oral minimal model, we observed a lower dynamic, static, and total insulin secretion in those with moderate and severe OSA (*p* < 0.0001, *p* = 0.007, *p* = 0.007, respectively) (Fig. 1, panels A–C).

**Fig. 2:**
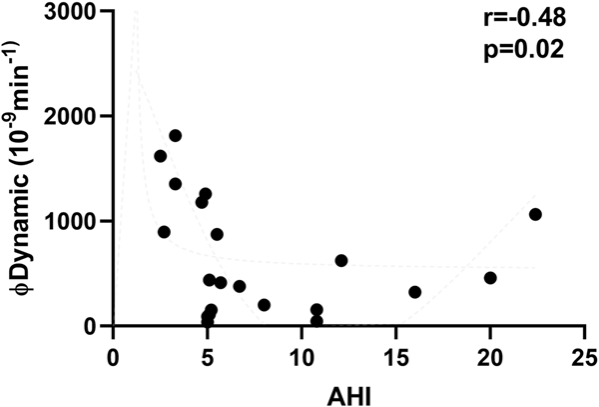
Spearman correlation between apnea/hypopnea index (AHI) and early insulin secretion phase. A significant negative correlation between AHI and dynamic insulin secretion was found (*r* = −0.48, *p* = 0.02).

Spearman correlation showed a negative correlation between AHI and φdynamic (*r* = −0.48, *p* = 0.02; Fig. 2). No correlation was however, found between the other polygraphy parameters, *φ*_total_ and *φ*_static_ (Supplementary Table 5). No gender effect was observed in our cohort.

## Discussion

Our study investigated global insulin secretion and the two different phases of beta-cell response after oral glucose in children and adolescents with obesity and OSA. The study conducted after standard 2-h OGTT revealed that children and adolescents with moderate or severe OSA displayed higher 30- and 60-min plasma glucose levels. In addition, they presented higher rates of elevated 1-hour plasma glucose that has been associated with lower insulin sensitivity and early insulin secretion in children and adolescents with obesity [16] . The association between 30-min plasma glucose and OSA severity was also verified by correlation and GLM analyses and it remained significant adjusting for confounding factors. By using the oral minimal model, we dissected the role of the single components of beta-cell responsiveness, showing that those with moderate and severe OSA have a lower dynamic and static component of beta-cell responsiveness as compared to those with mild OSA. As mentioned above, the dynamic component depends on pre-constituted insulin in pancreatic beta-cell and it influences early glucose raise during the OGTT. Among respiratory parameters, AHI correlated with late insulin secretion that describes de novo synthesis in pancreatic beta-cell. This secretion phase influences the late glucose plasma levels during OGTT and it’s impaired in IGT patients [23]. In contrast with previous studies, we found no differences in terms of insulin resistance measures between the groups.

This is the first study that performed oral minimal model to assess insulin secretion in pediatric OSA. OSA has been reported as an independent risk factor for T2D in adults and insulin resistance in both adults and children [4, 5, 8, 9, 24] However, the natural history of T2D development highlights the crucial role of beta-cell function impairment on diabetes occurrence [25]. Our results suggest an independent role for OSA in the natural history of T2D as we observed an impaired insulin secretion in children and adolescents with moderate and severe OSA. The pathogenic mechanisms underlying this association should be better investigated. OSA enhances oxidative stress and inflammation through chronic hypoxia and reoxygenation [22]. Animal models have demonstrated that chronic intermittent hypoxia damages pancreatic beta-cell through reactive oxygen species (ROS) production and inflammation [8]. The increased generation of ROS and the ensuing oxidative stress have been implicated in the progression of type 2 diabetes via pancreatic beta cell damage. Chronic intermittent hypoxia in mice models leads to persistent increase in mitochondrial ROS, which was due to impaired electron transport chain function at the complex I [8]. These processes impair glucose-induced insulin secretion [8, 9, 26]. and induce beta-cell apoptosis in rats [9]. Moreover, pancreatic inflammation, lesions and apoptosis, and insulin resistance worsened as oxygen saturation decreases, suggesting that the metabolic disorders and pancreatic injury rely on the OSA severity [9]. However, in our study, ODI, the main index of hypoxia-reoxygenation among respiratory parameters, was less associated with insulin secretion compared to AHI, suggesting that intermittent hypoxia is not the only responsible for beta-cell function derangement. Indeed, further pathophysiological mechanism can be advocated. For instance, sleep-breathing disorders have been associated with autonomic nervous system dysfunction, with a prevalence of sympathetic activity over parasympathetic system. Autonomic nervous system affects insulin sensitivity and secretion. Therefore, it might be hypothesized that this imbalance contributes to beta-cell dysfunction [27]. Moreover, it is likely that OSA-related sleep disruption, with consequent reduction of REM sleep stage with high rates of glucose utilization [28], places short sleepers at risk of poor glycemic control [28]. However, data about sleep architecture and autonomic function in our cohort are lacking. Therefore, to gain better knowledge of the complex OSA-glucose homeostasis relationship more studies should be conducted.

In addition to differences in insulin secretion, children and adolescents with severe OSA showed higher ALT levels compare to the other groups. This finding is in line to other scientific evidence that reported higher rates of non-alcoholic fatty liver disease (NAFLD) and elevated liver enzymes in children and adolescents with OSA compared to controls independent from confounding factors [29, 30]. In our cohort, we did not observe any gender effect on insulin secretion and glucose metabolism impairment. However, gender dimorphism in diabetes risk have been advocated [31]. This incongruence might be due to the small sample size that might underpower the study in identifying all the confounders.

We acknowledge that this study has some limitations. The main limitation is the lack of complete polysomnography involving EEG recordings; moreover, the subgroup undergoing the OMM is a small sample. In addition, the lack of information about adiposity distribution in the abdomen as well as the lack of information about percent body fat and fat free mass (FFM) limit the strength of the results. Despite that, it has to be taken into account that these studies (polygraphy and 3-h OGTT) are not easy to perform in young children. Further studies with larger sample size and longitudinal design might help to clarify the role of OSA in the natural history of type 2 diabetes and the potential role of non-invasive ventilatory support in restoring beta-cell function.

## Conclusions

Our study investigated the association between pediatric OSA and beta-cell function in children and adolescents with obesity. This is the first pediatric study that sought to assess whether the degree of OSA is associated with beta cell response and that has used the oral minimal model to estimate insulin secretion in pediatric OSA. Our findings support the previous data of increased risk of metabolic derangement in these patients. Moreover, our study suggests that sleep apnea may predispose to an early onset of prediabetes and type 2 diabetes by impairing early insulin secretion in children and adolescents with obesity.

## Supplementary information

Post-hoc analysis for differences in anthropometric, biochemical, and polysomnographic characteristics between mild, moderate, and severe OSA groups.

Logistic regression analysis for risk of showing elevated 1-h plasma glucose in the 2-h OGTT group

Differences in anthropometrical and clinical characteristics between 2-h OGTT and 3-h OGTT group.

Differences between mild, moderate, and severe OSA group of the 3-h OGTT test

Spearman correlation analysis between respiratory parameters and 3-h OGTT derived parameters.

## Data Availability

Data are available are available from the corresponding author on reasonable request.
