# Supplementary material for: Obstructive sleep apnea (OSA) is associated with the impairment of beta-cell response to glucose in children and adolescents with obesity
Source: Int J Obes (Lond). 2023 Jan 20;47(4):257–62. doi: 10.1038/s41366-023-01257-w (PMC10113157; doi:10.1038/s41366-023-01257-w)
Supplement: Supplementary file 1 — Post-hoc analysis for differences in anthropometric, biochemical, and polysomnographic characteristics between mild, moderate, and severe OSA groups. [file 41366_2023_1257_MOESM1_ESM.docx]

**Supplementary table 1.** Post-hoc analysis for differences in anthropometric, biochemical, and polysomnographic characteristics between mild, moderate, and severe OSA groups.

|  | P for difference | Mild vs Moderate OSA  P for difference | Mild vs Severe OSA  P for difference | Moderate vs Severe OSA  P for difference |
| --- | --- | --- | --- | --- |
| Age (years) | 0.08 | 0.39 | 0.10 | 0.31 |
| z-score BMI (years) | 0.17 | 0.99 | 0.19 | 0.46 |
| Fasting Glucose (mg/dL) | 0.74 | 0.99 | 0.99 | 0.99 |
| Fasting Insulin (mcU/mL) | 0.68 | 0.99 | 0.99 | 0.99 |
| 30-minutes glucose (mg/dL) | **0.01** | **0.01** | 0.11 | 0.99 |
| 60-minutes glucose (mg/dL) | **0.04** | **0.04** | 0.99 | 0.78 |
| 90-minutes glucose (mg/dL) | 0.83 | 0.99 | 0.99 | 0.99 |
| 120-minutes Glucose (mg/dL) | 0.32 | 0.99 | 0.99 | 0.40 |
| 120-minutes Insulin (µU/mL) | 0.23 | 0.26 | 0.13 | 0.99 |
| HOMA-IR | 0.81 | 0.99 | 0.99 | 0.99 |
| WBISI | 0.15 | 0.64 | 0.96 | 0.99 |
| IGI | 0.95 | 0.99 | 0.99 | 0.99 |
| DI | 0.53 | 0.41 | 0.99 | 0.99 |
| HDL-cholesterol (mg/dL) | 0.89 | 0.99 | 0.99 | 0.99 |
| Triglycerides (mg/dL) | 0.36 | 0.99 | 0.99 | 0.49 |
| LDL-cholesterol (mg/dL) | 0.53 | 0.99 | 0.91 | 0.99 |
| ALT (U/L) | 0.09 | 0.99 | 0.18 | 0.13 |
| AST (U/L) | 0.31 | 0.99 | 0.38 | 0.83 |
| AHI (events/hour) | **<0.0001** | **<0.0001** | **<0.0001** | **0.0008** |
| ODI (events/hour) | **<0.0001** | **0.004** | **<0.0001** | **0.0002** |
| Mean SpO2 (%) | **<0.0001** | 0.97 | **0.0009** | **0.0002** |
| Mean Desaturation (mean oxygen drop during events) | **0.0002** | 0.27 | **<0.0001** | **0.02** |
| Saturation nadir (%) | **<0.0001** | 0.68 | **<0.0001** | **0.001** |

Legend: AHI, apnea/hypopnea index; ALT, alanine aminotransferase; AST, aspartate aminotransferase; HOMA-IR, homeostasis model assessment for insulin resistance; IGI, insulinogenic index; DI, disposition index; ODI, oxygen desaturation index; WBISI, whole body insulin sensitivity index. Significant p values are reported in bold.
