# Supplementary material for: Obstructive sleep apnea (OSA) is associated with the impairment of beta-cell response to glucose in children and adolescents with obesity
Source: Int J Obes (Lond). 2023 Jan 20;47(4):257–62. doi: 10.1038/s41366-023-01257-w (PMC10113157; doi:10.1038/s41366-023-01257-w)
Supplement: Supplementary file 2 — Logistic regression analysis for risk of showing elevated 1-h plasma glucose in the 2-h OGTT group [file 41366_2023_1257_MOESM2_ESM.docx]

| Parameter | Odds Ratio (95% C.I.) | Unadjusted p | Adjusted Odds Ratio (95% C.I.) | Adjusted p |
| --- | --- | --- | --- | --- |
| Age | 1.1 (0.9-1.4) | 0.18 | 1.2 (0.8-1.9) | 0.35 |
| Z-score BMI | 1.1 (0.5-2.4) | 0.77 | 1.1 (0.4-2.8) | 0.84 |
| Gender | 0.7 (0.3-1.9) | 0.53 | 0.7 (0.2-2.6) | 0.64 |
| Tanner stage | 1.2 (0.4-3.3) | 0.75 | 1.4 (0.1-12.0) | 0.75 |
| OSA severity | 6.2 (1.6-23.4) | **0.007** | 11.5 (2.4-53.7) | **0.002** |

**Supplementary table 2.** Logistic regression analysis for risk of showing elevated 1-hour plasma glucose in the 2-hour OGTT group

The odd of showing elevated 1-hour plasma glucose is higher in children and adolescents with moderate and severe OSA compared to those with mild OSA independent of confounders. Unadjusted and adjusted odds ratio, 95% confidence intervals, and p level of significance are displayed. Statistical significant levels are reported in bold. Legend: OSA: obstructive sleep apnea. Pubertal status was defined according to Tanner stage evaluating breast development in girls and testicular volume and genitalia development in boys: prepubertal boys and girls were defined as Tanner I, post-pubertal boys and girls were defined as Tanner III.
