# Supplementary material for: Obstructive sleep apnea (OSA) is associated with the impairment of beta-cell response to glucose in children and adolescents with obesity
Source: Int J Obes (Lond). 2023 Jan 20;47(4):257–62. doi: 10.1038/s41366-023-01257-w (PMC10113157; doi:10.1038/s41366-023-01257-w)
Supplement: Supplementary file 3 — Differences in anthropometrical and clinical characteristics between 2-h OGTT and 3-h OGTT group. [file 41366_2023_1257_MOESM3_ESM.docx]

**Supplementary Table 3.** Differences in anthropometrical and clinical characteristics between 2-hour OGTT and 3-hour OGTT group.

|  | 2-hour OGTT  (N=55)  (%, or median and IQR) | 3-hour OGTT  (N=22)  (%, or median and IQR) | p for difference |
| --- | --- | --- | --- |
| Age (years) | 10.9 (8.9-14.0) | 12.3 (9.7-13.6) | 0.76 |
| Gender (M) | 43.6 | 54.6 | 0.37 |
| Tanner stage (I/II/III) | 47.3/27.3/25.4 | 33.3/33.3/33.4 | 0.68 |
| Z-score BMI | 3.5 (3.1-3.7) | 3.6 (3.2-3.9) | 0.37 |
| OSA severity (mild, moderate, severe) | 40.0/36.4/23.6 | 31.8/40.9/27.3 | 0.80 |

Legend: OGTT: oral glucose tolerance test; OSA: obstructive sleep apnea. Pubertal status was defined according to Tanner stage evaluating breast development in girls and testicular volume and genitalia development in boys: prepubertal boys and girls were defined as Tanner I, post-pubertal boys and girls were defined as Tanner III.
