# Supplementary material for: Obstructive sleep apnea (OSA) is associated with the impairment of beta-cell response to glucose in children and adolescents with obesity
Source: Int J Obes (Lond). 2023 Jan 20;47(4):257–62. doi: 10.1038/s41366-023-01257-w (PMC10113157; doi:10.1038/s41366-023-01257-w)
Supplement: Supplementary file 4 — Differences between mild, moderate, and severe OSA group of the 3-h OGTT test [file 41366_2023_1257_MOESM4_ESM.docx]

**Supplementary table 4.** Differences between mild, moderate, and severe OSA group of the 3-hour OGTT test

|  | Mild OSA  (n=7)  (%, or median and IQR) | Moderate OSA  (n=9)  (%, or median and IQR) | Severe OSA  (n=6)  (%, or median and IQR) | P for difference |
| --- | --- | --- | --- | --- |
| Gender (M) | 50 | 48 | 49 | >0.99 |
| Age (years) | 12.8 (10.0-13.6) | 9.4 (8.4-11.2) | 10.4 (9.4-11.2) | 0.26 |
| Tanner stage  (I, II, III) | 43.3/33.3/23.4 | 66.7/16.7/16.6 | 56.5/26.7/16.8 | 0.25 |
| BMI z-score | 3.3 (I3.1-3.5) | 3.9 (3.7-4.2) | 4.0 (3.6-4.2) | 0.13 |
| Fasting Glucose (mg/dL) | 69 (64-71) | 65 (57-71) | 68 (60-71) | 0.47 |
| Fasting Insulin (mcU/mL) | 20.7 (19.2-38.3) | 15.1 (9.5-33.7) | 18.1 (11.5-35.7) | 0.40 |
| 30-minutes glucose (mg/dL) | 118 (112-118) | 135 (128-155) | 137 (120-145) | 0.10 |
| 60-minutes glucose (mg/dL) | 123 (106-126) | 145 (113-157) | 148 (120-159) | 0.23 |
| 120-minutes glucose (mg/dL) | 109 (98-123) | 95 (76-108) | 155 (120-179) | 0.20 |
| 120-minutes Insulin (mcU/mL) | 130.8 (45.2-196.9) | 47.5 (16.2-108) | 87.5 (56.2-148) | 0.10 |
| HOMA-IR | 2.3 (2.0-6.5) | 3.3 (1.7-5.3) | 3.6 (1.9-5.8) | 0.23 |
| WBISI | 3.0 (2.1-3.6) | 3.4 (1.7-6.1) | 2.6 (1.8-4.0) | 0.59 |
| IGI | 1.5 (1.2-2.1) | 1.9 (0.9-2.4) | 1.7 (1.1-2.7) | 0.58 |
| DI | 4.4 (2.6-4.3) | 4.2 (3.0-6.8) | 4.1 (3.3-5.8) | 0.99 |
| HDL-cholesterol (mg/dL) | 35 (32-42) | 39 (36-45) | 37 (31-44) | 0.47 |
| Triglycerides (mg/dL) | 116 (92-152) | 148 (92-252) | 128 (88-182) | 0.75 |
| ALT (U/L) | 36 (22-65) | 26 (21-45) | 29 (20-40) | 0.69 |
| AST (U/L) | 24 (18-39) | 24 (22-32) | 24 (21-30) | 0.52 |

Legend: ALT, alanine aminotransferase; AST, aspartate aminotransferase; HOMA-IR, homeostasis model assessment for insulin resistance; IGI, insulinogenic index; DI, disposition index; WBISI, whole body insulin sensitivity index. Pubertal status was defined according to Tanner stage evaluating breast development in girls and testicular volume and genitalia development in boys: prepubertal boys and girls were defined as Tanner I, post-pubertal boys and girls were defined as Tanner III.
