# Supplementary material for: Obstructive sleep apnea (OSA) is associated with the impairment of beta-cell response to glucose in children and adolescents with obesity
Source: Int J Obes (Lond). 2023 Jan 20;47(4):257–62. doi: 10.1038/s41366-023-01257-w (PMC10113157; doi:10.1038/s41366-023-01257-w)
Supplement: Supplementary file 5 — Spearman correlation analysis between respiratory parameters and 3-h OGTT derived parameters. [file 41366_2023_1257_MOESM5_ESM.docx]

|  | AHI | ODI | Saturation nadir | Mean oxygen desaturation | Mean SpO2 | φdynamic | φstatic | DI | φtotal | SI |
| --- | --- | --- | --- | --- | --- | --- | --- | --- | --- | --- |
| AHI | 1.000 | 0.83*** | -0.30 | 0.57** | -0.23 | -0.48 | -0.31 | 0.12 | -0.28 | 0.31 |
| ODI |  | 1.000 | -0.33 | 0.46* | -0.27 | -0.34 | -0.21 | 0.11 | -0.18 | 0.21 |
| Saturation nadir |  |  | 1.000 | -0.83*** | 0.27 | -0.19 | -0.31 | -0.55** | -0.36 | -0.30 |
| Mean oxygen desaturation |  |  |  | 1.000 | -0.15 | -0.16 | 0.06 | 0.60** | 0.06 | 0.54** |
| Mean SpO2 |  |  |  |  | 1.000 | -0.20 | -0.47* | -0.21 | -0.33 | 0.02 |
| φdynamic |  |  |  |  |  | 1.000 | 0.79*** | 0.27 | 0.80*** | -0.29 |
| φstatic |  |  |  |  |  |  | 1.000 | 0.33 | 0.89*** | -0.26 |
| DI |  |  |  |  |  |  |  | 1.000 | 0.48* | 0.68** |
| φtotal |  |  |  |  |  |  |  |  | 1.000 | -0.20 |
| SI |  |  |  |  |  |  |  |  |  | 1.000 |

**Supplementary table 5.** Spearman correlation analysis between respiratory parameters and 3-hour OGTT derived parameters.

Correlations between variables were assessed by Spearman correlations, Spearman’s r coefficient is displayed for each correlation test. Significance level is expressed as follows: *p<0.05; **p<0.01; ***<0.0001.

Legend: AHI, apnea/hypopnea index; DI, disposition index; ODI, oxygen desaturation index; Mean SpO2: mean oxygen saturation; saturation nadir: lowest oxygen saturation; SI: insulin sensitivity; φdynamic: dynamic insulin secretion; φstatic: static insulin secretion; φtotal: total insulin secretion;
